# Engrafted newborn neurons could functionally integrate into the host neuronal network

**DOI:** 10.13918/j.issn.2095-8137.2017.005

**Published:** 2017-01-18

**Authors:** Zheng-Bo Wang, Dong-Dong Qin, Xin-Tian Hu

**Affiliations:** ^1^ Key Laboratory of Animal Models and Human Disease Mechanisms of Chinese Academy of Sciences & Yunnan Province, Kunming Institute of Zoology, Kunming Yunnan 650223, China; ^2^CAS Center for Excellence in Brain Science and Intelligence Technology, Chinese Academy of Sciences, Shanghai 200031, China

The limited capability to regenerate new neurons following injuries of the central neural system (CNS) still remains a major challenge for basic and clinical neuroscience. Neural stem cells (NSCs) could nearly have the potential to differentiate into all kinds of neural cells *in vitro*. Previous studies verified that exogenous transplanted NSCs are capable of differentiating into neurons and projecting onto the host neurons in the rat brain (Tabar et al., 2005; Dong JR et al., 2012), which could lead to behavioral recovery from neuronal damages such as spinal cord injury (McDonald et al., 1999), Parkinson's disease (Gonzalez et al., 2015; Kim et al., 2002; Lindvall, 2001), and stroke (Zhang et al., 2016). In January, 2013, the Food and Drug Administration (FDA) of United States has given Neuralstem clearance to commence its human spinal stem cell transplantation in chronic spinal cord injury (Orelli, 2013). In addition, this company is evaluating the safety of stem cell transplantation for the treatment of major depressive disorder (MDD), chronic traumatic encephalopathy (CTE), Alzheimer's disease, and post-traumatic stress disorder (PTSD) (Neuralstem Inc., 2013). However, the mechanism underlying this treatment is controversial. It is still unknown whether the secretion of trophic factors by the transplanted NSCs slowed or prevented the deterioration of the degenerating neurons (Breunig et al., 2011; Xiao et al., 2015; Zuo et al., 2015), or the new neurons differentiated from the transplanted NSCs substituted the injured or lost neurons by functionally integrating into the host neural circuitry (Englund et al., 2002; Weick et al., 2011). To illustrate this question and find out how likely the injured neurons were substituted with new neurons, the functional examination is preferentially to be carried out on an intact animal model (or awake animal). Generally, stem cell clusters transplanted into the brain parenchyma will migrate as single cells towards diverse brain regions. In view of this, approaches that could lock the engrafted cell mass in the specific site were required (Yang SC et al., 2011). Herein, a new technique was developed where a small 'hole' was created in the inferior colliculus (IC) of rhesus monkeys to lock the transplanted NSCs in situ and investigate their integration into the host auditory neural network. The results showed that a substantial portion of transplanted cells differentiated into mature neurons and formed synaptic input/output connections with the host neurons. Under awake condition, c-fos expression in newborn neurons was found to be significantly increased after acoustic stimulation and multichannel recordings indicated IC specific tuning activities in response to auditory stimulation were also recorded in newborn neurons as the reported IC neurons (Zwiers et al., 2004). These results suggest that the transplanted cells have functionally integrated into the host neural network in the awake monkey brain and provided a strong foundation for the future stem cell treatment of the CNS injuries (Wei et al., 2016).

This is the first time evaluating the neurons differentiated from the transplanted NSCs in awake animal, which was further confirmed by Mark Hübener and his group working at the Max Planck Institute of Neurobiology (Falkner et al., 2016). They also found that the transplanted neural stem cells could differentiate into local neuronal phenotype, target projection and functionally integrate with the host neurons. Both of the findings revealed that engrafted stem cells could differentiate into mature neurons, form synaptic with the host neurons, successfully substitute the injured or lost neurons and functionally integrate with the host neuronal network, which might be necessary for the normal brain function of awake animal. 

The current findings provide impetus for stem-cell therapy, but if you want to fully assess the ability of transplanted stem cells to differentiate and replace lost neurons in a damaged brain, further sophisticated experimentation should be conducted. For example, the high-throughput dual-color precision imaging (Gong et al., 2016) or transparent intact brain technique (Ke et al., 2013; Susaki et al., 2014; Yang et al., 2014) can be used to reconstruct the engrafted neurons' projections with the host, from which we can overview the newborn neurons and their integration with the host from a systems perspective. All in all, these findings provide encouragement that stem cell replacement therapy will become a reality in the near future.
